# Urofacial Syndrome, an Expressive and Unmasking Sign of Voiding Dysfunction: Case Report and Review of the Literature

**DOI:** 10.1155/criu/5548217

**Published:** 2025-09-05

**Authors:** Fawaz Alkeraithe, Waleed Altulayqi, Mutasim Alkhalifah, Faisal Alasmari, Mohammad Asiri, Ahmad Alhazmi

**Affiliations:** Department of Urology, King Fahad Medical City, Riyadh, Saudi Arabia

**Keywords:** dysfunction, facial, inverted, neuromodulation, Ochoa, sacral

## Abstract

Urofacial syndrome (UFS) is a rare autosomal recessive disorder characterized by voiding dysfunction, inverted facial expressions when smiling, and potential mutations in LRIG2 or HPSE2 genes. We report two sisters diagnosed with UFS who were managed using sacral neuromodulation (SNM). The first, aged 24, had recurrent UTIs, chronic urinary retention, and a trabeculated bladder without vesicoureteral reflux (VUR). Genetic testing identified a homozygous LRIG2 variant. Following SNM, her voiding function improved, reducing the frequency of catheterization. The second patient, aged 27, presented with a Grade 5 left-sided VUR, severe hydronephrosis, and a nonfunctioning left kidney. Urodynamic studies revealed an acontractile bladder. Post-SNM, her postvoid residual decreased to 30 mL, allowing independent voiding. Both displayed the hallmark inverted facial grimace. Diagnostic imaging and urodynamics confirmed neurogenic bladder, excluding spinal anomalies. Management included clean intermittent catheterization (CIC) and SNM, which enhanced bladder emptying and reduced catheter dependence. This case highlights SNM as a promising therapeutic option in UFS, improving voiding efficiency and quality of life. Early recognition of the distinctive facial expression is critical to prevent upper tract damage. Urologists should suspect UFS in patients with voiding dysfunction and abnormal facial expressions, considering SNM as a viable intervention. Further studies on SNM's role in UFS are warranted.

## 1. Introduction

Urofacial syndrome (UFS), also known as Ochoa syndrome, is a rarely seen genetic disorder with an inheritance pattern of autosomal recessive. Approximately 150 cases have been reported in the literature. The syndrome was first described by Dr. Bernardo Ochoa, when he encountered a patient with end-stage chronic kidney disease secondary to bladder dysfunction and vesicoureteral reflux (VUR) along with an abnormal facial expression in 1964 [1]. Affected individuals commonly experience urinary voiding difficulties, such as incomplete bladder. Some may also present with a confirmed genetic mutation and a characteristic inverted facial expression; these patients appear sad or as if they are crying when they try to smile [2]. In this paper, we report two sisters with UFS who were successfully managed with sacral neuromodulation (SNM).

## 2. Case Presentation 1

**Figure 1:**
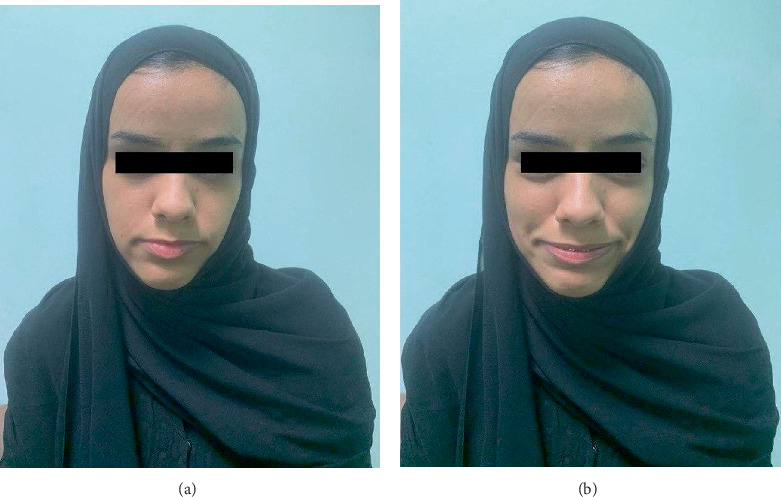
(a) Facial expression without smile. (b) Inverted facial expression when she smiles.

**Figure 2:**
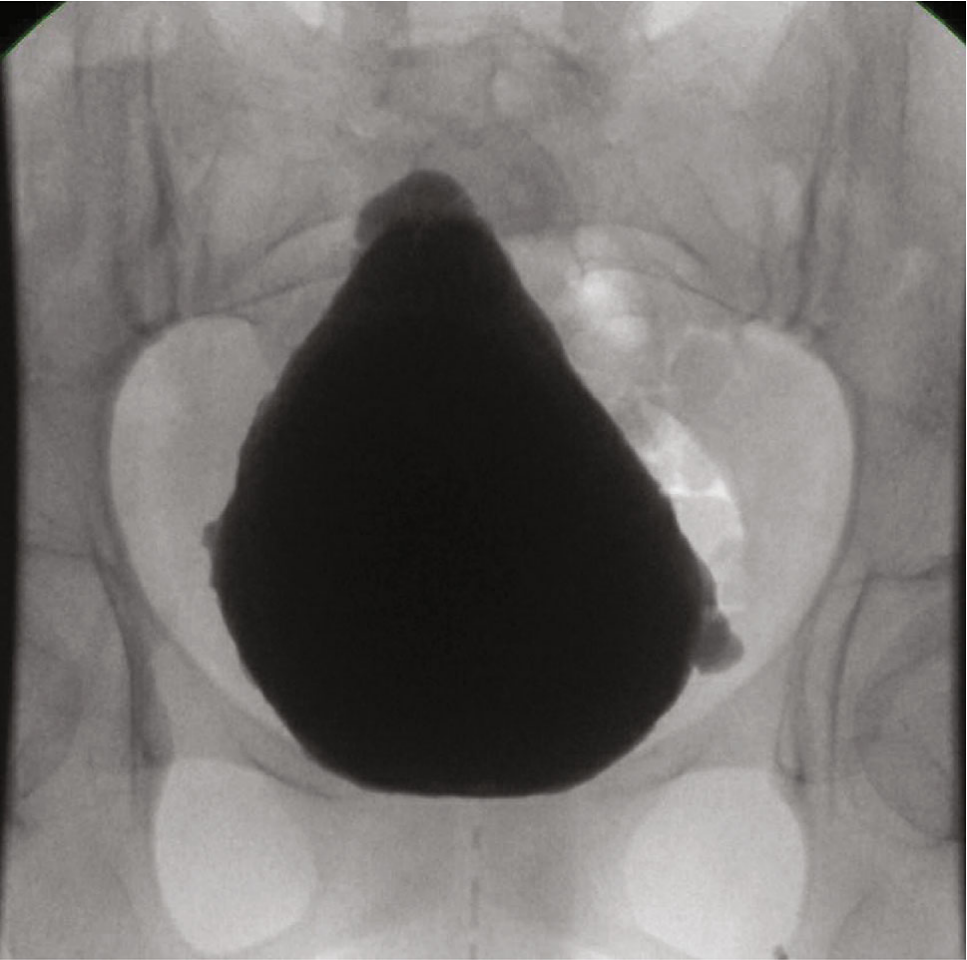
The trabeculated bladder wall with few diverticular *outpouchings* exhibiting a Christmas tree appearance.

A 24-year-old female presented with a history of recurrent urinary tract infection (UTI) and chronic urinary retention since childhood. She exhibited a distinct inverted facial expression when smiling which affected her social life (Figure 1a,b). Creatinine level is 0.54 mg/dL. An ultrasound (US) of the kidneys and bladder shows signs of neurogenic bladder with irregular bladder wall without associated hydronephrosis on either side. Fluoroscopic cystogram revealed a trabeculated wall with few diverticular outpouchings showing a Christmas tree appearance. No VUR was seen (Figure 2). Magnetic resonance imaging (MRI) of the spine was unremarkable. Urodynamic study showed small bladder capacity with mild detrusor contraction during filling without leak and being unable to void without a catheter. The patient was managed with clean intermittent catheterization (CIC) every 4 h and was counseled about SNM. During the first stage of insertion, intraoperatively, the lead was placed at S3 and showed good pelvic floor muscle contraction (pelvic floor bellows), along with the flexion of the big toe, at a setting of 1.5 across four points. Her urination was improved with the device, and she was able to pass urine on her own, reducing the need for CIC use to twice a day. A genetic testing revealed homozygous variant at the LRIG2: NM_014813.2: c.1421C>G, p.Pro474Arg, which was a type of variation called a missense change. A follow-up family test confirmed the diagnosis of UFS Type 2, which is passed down in families in autosomal recessive pattern.

## 3. Case Presentation 2

**Figure 3:**
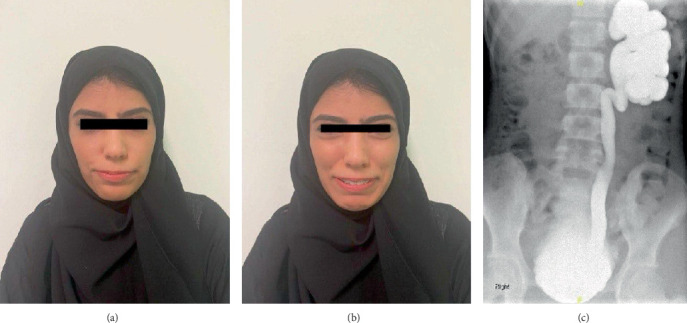
(a) Facial expression without smile. (b) Inverted facial expression when she smiles. (c) Left-sided VUR Grade V.

**Figure 4:**
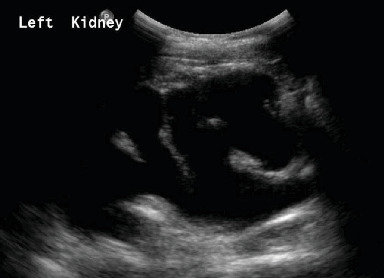
Severe left hydronephrosis with thinning of the parenchyma.

A 27-year-old female patient, whose parents are first cousins, was referred to the outpatient department for recurrent UTIs, chronic retention, and occasional left flank pain. On physical examination, she has inverted facial expression that is noticed when she smiles (Figure 3). Creatinine level was 0.5 mg/dL. The voiding cystourethrogram (VCUG) showed a thickened, trabeculated bladder with irregular outline. Moreover, it has left Grade 5 VUR, and the patient was unable to void at the end of the study (Figure 3). The US of the kidneys and bladder showed severe left hydronephrosis with thinning of the parenchyma, right mild hydronephrosis, and a distended bladder with an irregular outline which is suggestive of neurogenic bladder (Figure 4). The dimercaptosuccinic acid scan showed a compensatory hypertrophied right kidney with good function and a nonfunctioning left kidney. Urodynamic study indicated an acontractile bladder with complete failure. Her spine MRI was unremarkable. As a consequence, the patient was started on CIC every 4 h and antibiotic for her recurrent UTIs. The patient was counseled regarding the trial of SNM device insertion, and she agreed. During the first stage of insertion, intraoperatively, the lead was placed at S3, resulting in good pelvic floor muscle contraction (pelvic floor bellows) along with the flexion of the big toe at a setting of 1.5 across four points. The patient underwent the first-stage trial for 2 weeks during which she was able to urinate on her own. After urination, using CIC, the maximum amount reached 30 mL, so a decision was made to proceed with the second stage of SNM which was placed without complications. During the investigation, the patient was referred to the genetic services, and genetic testing revealed a homozygous variant at the LRIG2: NM 014813.2: c.1421C>G, p.Pro474Arg, which was classified as a homozygous, missense VUS, and segregation test was conducted which confirmed the diagnosis with an autosomal recessive UFS Type 2.

## 4. Discussion

Dr. Ochoa was the first to identify the association between abnormal facial expression and diurnal or nocturnal enuresis in patients with recurrent UTI [1]. Three components have been described for UFS that are not necessarily always present for the diagnosis: voiding or defecation dysfunction, inverted facial expression, and genetic component. The inverted facial expression that is a unique sign of UFS is not a structural facial defect, but it is more of dysfunctional expression. These people seem sad when they smile. However, they have the normal facial expression when they are sad [2]. UFS follows an autosomal recessive inheritance pattern. Two genes have been identified in the association of the syndrome: biallelic mutations in the HPSE2 genes (inactive heparanase-2) located on chromosome 10q23-q24 and the LRIG2 gene (leucine-rich repeats and immunoglobulin-like domain Protein 2) located on chromosome 1p13.2. However, some have been identified to be the possible causative genes in UFS, although some patients with UFS have no genetic mutation [3].

Ochoa studied 50 patients with UFS and reported that 100% of them had a trabeculated bladder wall on VCUG, 64 had VUR, and 78% had varying degrees of renal scarring. In addition, 24% (*n* = 12) of them were in chronic kidney disease, and nine of them ended up with end-stage renal disease. Hydronephrosis can give a hint, and it is present in 58% of patients [4]. Similarly, this patient has severe hydronephrosis which could potentially put the kidney at risk. Alqasem et al. reported a case of UFS in a 7-year-old Saudi boy with all three components: inverted facial expression, recurrent urinary retention, and confirmed genetic testing of UFS. VUR and a trabeculated bladder wall seem to be common findings, as observed in our case and in the case series by Ochoa [4, 5].

UFS should be in the differential diagnosis for urologist who evaluate a patient with inverted facial expression in addition to sign and symptoms of voiding dysfunction. Short- and long-term management should focus on preserving renal function and preventing further renal deterioration, as well as ensuring effective bladder emptying through CIC, combined with the judicious use of antibiotics and anticholinergics. For pediatric patients whose families cannot perform CIC, a vesicostomy can be considered a temporary option until the patient shows competency and dependency to use CIC by their own [4]. As SNM is approved for refractory nonneurogenic atonic bladder, we elected to proceed with SNM trial implantation for both patients. Each exhibited chronic urinary retention necessitating intermittent catheterization due to persistent failure of free void. This intervention is aimed at restoring bladder function, eliminating catheter dependence, and improving long-term quality of life and clinical outcomes.

## 5. Conclusion

Early recognition of UFS is crucial to prevent progressive damage to the upper urinary tract. A simple examination of facial expression can lead to timely diagnosis. Urologist should investigate thoroughly any patient who presents with inverted facial expression and voiding dysfunction. Urologists are encouraged to report clinical features and treatment methods, especially emerging therapy of neuromodulation in such patients.

## Data Availability

The data that support the findings of this study are available from the corresponding author upon reasonable request.

## Nomenclature

UFS: urofacial syndrome
UTI: urinary tract infection
VUR: vesicoureteral reflux
CIC: clean intermittent catheterization
SNM: sacral neuromodulation
MRI: magnetic resonance imaging
VCUG: voiding cystourethrogram
DMSA: dimercaptosuccinic acid
